# Athletic Injury Research: Frameworks, Models and the Need for Causal Knowledge

**DOI:** 10.1007/s40279-024-02008-1

**Published:** 2024-03-20

**Authors:** Judd T. Kalkhoven

**Affiliations:** 1 School of Health Sciences, Western Sydney University, Campbelltown, NSW Australia; 2 Human Performance Research Centre, Faculty of Health, University of Technology Sydney, Sydney, NSW Australia

## Abstract

Within applied sports science and medicine research, many challenges hinder the establishment and detailed understanding of athletic injury causality as well as the development and implementation of appropriate athletic injury prevention strategies. Applied research efforts are faced with a lack of variable control, while the capacity to compensate for this lack of control through the application of randomised controlled trials is often confronted by a number of obstacles relating to ethical or practical constraints. Such difficulties have led to a large reliance upon observational research to guide applied practice in this area. However, the reliance upon observational research, in conjunction with the general absence of supporting causal inference tools and structures, has hindered both the acquisition of causal knowledge in relation to athletic injury and the development of appropriate injury prevention strategies. Indeed, much of athletic injury research functions on a (causal) model-blind observational approach primarily driven by the existence and availability of various technologies and data, with little regard for how these technologies and their associated metrics can conceptually relate to athletic injury causality and mechanisms. In this article, a potential solution to these issues is proposed and a new model for investigating athletic injury aetiology and mechanisms, and for developing and evaluating injury prevention strategies, is presented. This solution is centred on the construction and utilisation of various causal diagrams, such as frameworks, models and causal directed acyclic graphs (DAGs), to help guide athletic injury research and prevention efforts. This approach will alleviate many of the challenges facing athletic injury research by facilitating the investigation of specific causal links, mechanisms and assumptions with appropriate scientific methods, aiding the translation of lab-based research into the applied sporting world, and guiding causal inferences from applied research efforts by establishing appropriate supporting causal structures. Further, this approach will also help guide the development and adoption of both relevant metrics (and technologies) and injury prevention strategies, as well as encourage the construction of appropriate theoretical and conceptual foundations prior to the commencement of applied injury research studies. This will help minimise the risk of resource wastage, data fishing, p-hacking and hypothesising after the results are known (HARK-ing) in athletic injury research.

## Key Points

**Table:** 

Athletic injury research has a large reliance upon observational research to guide applied practice. However, appropriate supporting causal inference tools and structures are generally absent within the existing literature, which is problematic.
Owing to various forms of bias, such as confounding and collider-stratification bias, current research approaches may erroneously implicate variables that are protective to athletes with an unchanged or increased injury risk, as well as variables that are harmful to athletes with an unchanged or decreased injury risk. This may facilitate the implementation of injury prevention strategies that are ineffective or, worse, increase the risk of injury and interfere with an athlete’s training process.
To help alleviate these concerns, athletic injury research and prevention efforts should shift their attention towards the formation, utilisation, investigation and when necessary, revision or replacement of causal diagrams including theoretical and conceptual causal frameworks and models, DAGs and similar diagrammatical constructs.

## Introduction

Injuries in sport remain a major concern to athletes, practitioners and sporting organisations as the negative impacts of athletic injuries are widespread, affecting athlete health, individual and team performances, as well as sporting clubs’ economies. Additionally, previous injury remains one of the leading risk factors for an increased risk of subsequent injury [1, 2], although in some contexts the causal nature of this relationship has been questioned [3]. Large scale epidemiological studies continue to report the persistent nature of athletic injuries, with injury rates remaining relatively stable over a number of years across a multitude of sports [4–6]. Markedly, some types of injuries, such as hamstring injuries, are reportedly even increasing in certain contexts [7, 8].

Although it is difficult to attribute any lack of progress relating to athletic injury prevention to a single factor, a potential major contributor is a general absence of understanding regarding athletic injury causality. Indeed, injury prevention strategies have been widely implemented in the absence of causal explanations, causal frameworks or more generally, coherent supporting theories and conceptual foundations [9–11]. Such strategies are contradictory to the popularised ‘sequence of prevention’ [12], whereby the establishment of injury causality and aetiology form the foundations on which appropriate injury prevention strategies should be developed [12].

Determining the causes of natural phenomena is a key goal of science [13], and is an a priori requirement to the manipulation of variables to produce a favourable outcome. However, in many scientific areas, developing causal knowledge is difficult. Within applied sports science and medicine research, numerous challenges hinder attempts to develop detailed understandings of injury causation. For example, the application of randomised controlled trials (RCT; Table 1), particularly in elite sporting populations, is often confronted by a number of obstacles relating to either ethical or practical constraints that restrict their implementation. Such difficulties have arguably resulted in poor research practices and an over-reliance on observational research (Table 1) (without appropriate supporting causal inference tools and structures) for guiding both the acquisition of causal knowledge and the development and implementation of athletic injury prevention strategies. In addition, many current approaches to athletic injury research and prevention, and the development of new metrics in this area, appear to be primarily driven by the mere existence and availability of various technologies and data. For example, many metrics utilising technologies, such as global positioning systems (GPS) or inertial measurement units (IMU), have been proposed and adopted across the research and sporting landscape to assess athletic injury risks. However, little attention has been devoted to how these metrics and their underlying technologies can conceptually be related to the causal (mechanical and physiological) processes and mechanisms governing athletic injury occurrence (of which many different types exist) [9–11]. Such approaches are not reflective of theory-driven research, which is an essential component of the scientific method [14, 15]. Rather, these approaches more closely resemble a (causal) model-blind approach at high risk of bias, data fishing [16], p-hacking [16, 17] and hypothesising after the results are known (HARK-ing) [16, 18, 19]. Considering the current reliance on observational studies in athletic injury research, there remains an uncomfortable scarcity of much needed supporting causal inference tools and structures within the existing literature to guide statistical analyses and causal inferences. It follows that, many current approaches to understanding athletic injury and developing injury prevention strategies in the applied sporting world lack causal justification and are overly speculative.

**Table 1:** Relevant nomenclature

Operational definitions	
Randomised controlled trial (RCT)	An RCT is a type of scientific experiment that aims to reduce certain sources of bias when testing the effectiveness of new treatments or interventions. This is achieved by randomly assigning participants to either the treatment group or the control group, with the randomisation process creating an expectation of ‘no confounding’ and exchangeability between groups
Observational research	Observational research is a research method in which the investigator does not intervene or manipulate the study environment or subjects but rather observes and measures variables of interest without altering their natural state. This approach is used to identify and describe patterns, trends and relationships within data
Exchangeability	Refers to the assumption that individuals or groups being compared in a study are interchangeable or equivalent with respect to all factors that cause the outcome, except for the variables or interventions under investigation. This assumption is crucial for the validity of RCTs and for making valid causal inferences, and ensures that any differences observed can be attributed to the specific exposure or treatment being studied rather than differences in baseline characteristics
Internal validity	Internal validity refers to the degree to which a study accurately establishes a causal relationship between variables; specifically, the extent to which it can be confidently stated that the change in the dependent variable was produced solely by the independent variable and not by any other factors
External validity	External validity refers to the extent to which the results of a study can be applied to other situations, settings, populations or time periods beyond the original conditions of the study
Transportability	Transportability is a more specific term than external validity, and refers to the extent to which causal conclusions from one study conducted in one population can be applied to a different external target population. This is in contrast to generalisability, which refers to the extent to which findings from a study conducted with a specific sample can be applied to the broader population from which the sample was drawn
Causal directed acyclic graph (DAG)	A causal DAG is a graphical tool used to represent causal relationships. In a DAG, nodes represent variables (such as factors or outcomes), and directed edges (arrows) represent causal influences from one variable to another. The ‘acyclic’ aspect means that the graph does not contain any cycles, implying that causality is not circular
Clinical equipoise	Clinical equipoise refers to a genuine uncertainty within the expert medical community about the comparative therapeutic merits of each arm of a clinical trial. It is an ethical precondition for the justification of conducting a RCT, ensuring that no patient is knowingly given an inferior treatment
Confounding	Confounding occurs when a third variable, known as a confounder, affects both the independent variable (the cause or treatment being studied) and the dependent variable (the outcome or effect being measured), leading to a spurious (non-causal) association between these two variables. This phenomenon leads to a false estimation of the causal effect of one variable on another
Confounder	A confounder is a variable that serves as a common cause of both the dependent and independent variables, creating a spurious association between them. This can result in misleading conclusions about the relationship between the variables being studied. Properly identifying and controlling for confounders is crucial in statistical analysis to accurately determine causal relationships
Deconfounder	Refers to a variable or a set of variables that, when controlled for, can help to reduce or eliminate confounding bias
Stress	Stress is defined as force per unit area and develops within a structure/tissue in response to an applied force. Stress is descriptive of the internal forces neighbouring particles of a given material exert on one another. Stress may be characterised as normal (force perpendicular to a plane) or shear (force parallel to a plane). Normal stress may be tensile or compressive depending on the mode of loading
Strain	Refers to the amount of deformation expressed as a normalized change in shape or size. Two basic types of strain exist: normal strain, which is related to change in length, and shear strain, which is related to change in angle. Normal strain is the ratio of deformation (lengthening or shortening) to original length and as such may be tensile or compressive. Shear strain is the amount of angular deformation that occurs in a structure. For example, a rectangle drawn on one face of a solid before a shear stress is applied will appear as a parallelogram during the application of a shear stress
Mechanical strength	Mechanical strength refers to the ability of a tissue to withstand and resist applied forces or loads without undergoing significant breakage or failure. It is a measure of how much stress and strain a tissue can handle before it begins to break or fail (athletic injury in this context)
Theory	In essence, a scientific theory is an explanation of a phenomenon in the natural world. It is used to make predictions that are testable by experiments or observations
Theoretical framework	Theoretical frameworks are structures that guide research by relying on a formal theory; that is, the framework is constructed by using an established, coherent theory (explanation) of certain phenomena and relationships
Practical framework	Practical frameworks are structures informed by the accumulated practical knowledge (ideas) of practitioners. In this respect, practical frameworks rely on conventional wisdom including commonly held beliefs, opinions and anecdotal experience. While this is a feature of this particular kind of framework, it also makes this type of framework particularly susceptible to bias
Conceptual framework	A conceptual framework refers to a compilation of concepts and/or constructs that are organised systematically to provide a foundation and tool for integrating and interpreting knowledge on a particular topic
Mediation	Mediation refers to the process through which an independent variable influences an outcome variable indirectly through one or more intervening variables, known as mediators. These mediators help to explain the mechanism or pathway by which the initial variable exerts its effects on the outcome, providing insights into the underlying causal chain
Mediator	A mediator is a variable that lies in the causal pathway between an independent variable and a dependent variable. It represents the mechanism that transmits the effect of one variable on another
Back-door path	In a DAG, a back-door path is any path from the treatment (or exposure) variable to the outcome variable that goes through a common cause or confounder. Unlike a direct path, which represents a hypothesised causal effect, a back-door path indicates a non-causal association that can produce a spurious correlation between the treatment and outcome
Causal mediation analysis	Causal mediation analyses use statistical methods to examine how an independent variable influences an outcome through one or more mediator variables, distinguishing the effects into direct and indirect (mediated) pathways
Direct effect	In causal inference, the direct effect refers to the impact of one variable on another without any intermediate variables mediating the relationship. It represents the direct causal pathway from the cause to the effect
Indirect effect	The indirect effect, also known as the mediated effect, is the influence of one variable on another through one or more intermediate variables in a causal pathway. It represents the causal effect that is transmitted through one or more mediators
Collider	A collider is a type of variable that is influenced by two or more other variables in a causal diagram or model. When two variables both influence a third variable (the collider), conditioning on this collider can create a spurious (non-causal) association between the two influencing variables, even if they are otherwise independent. This can lead to biased results in statistical analyses, making it crucial to identify and appropriately handle colliders in causal studies
Collider-stratification bias	Collider-stratification bias is a specific type of bias that occurs when the researcher (1) conditions on a collider variable or (2) stratifies their analysis on the basis of it
Sufficient causal set	A sufficient causal set is a specific combination of factors that, when present together, are sufficient to cause an outcome

To assist with some of the concerns described above, causal diagrams, including frameworks [20], models [14, 21], causal directed acyclic graphs (DAGs; Table 1) [22–26] and other causal diagrams [27–29], are relevant tools that provide substantial value, organising ideas and directing future research and causal inferences. Specifically, these tools help guide the research process by outlining key concepts relating to causality, such as relevant causal assumptions, pathways and mechanisms. In addition, these diagrams also have important implications for statistical analyses, with causal DAGs in particular outlining which variables to include and adjust for in a statistical analysis to identify causal and non-causal effects. Indeed, causal DAGs are instrumental in addressing bias, explicitly illustrating potential confounders (Table 1), while also highlighting when controlling for a variable inappropriately will introduce new bias into the analysis, such as collider-stratification bias (Table 1). Accordingly, the adoption of causal diagrams may help alleviate many of the challenges facing athletic injury research by facilitating the investigation of specific causal links, assumptions and mechanisms with appropriate scientific methods, aiding the translation of lab-based research into the applied sporting world, and guiding causal inferences from applied research efforts by establishing appropriate supporting causal structures. Further, the utilisation of causal diagrams will also help with the development and adoption of both relevant metrics (and technologies) and appropriate injury prevention strategies, and encourage the construction of coherent theoretical and conceptual foundations prior to the commencement and allocation of resources to applied injury research efforts. This will help minimise the risk of bias, resource wastage, data fishing [16], p-hacking [16, 17] and HARK-ing [16, 18, 19] in athletic injury research. In light of these proposed benefits of causal diagrams, the aims of this review article are two-fold; (1) to highlight some of the major challenges and shortcomings of some currently adopted approaches to athletic injury research, and (2) to illustrate how using causal diagrams for athletic injury research can lead to more robust scientific findings and causal understandings. Further, within this article a new model proposing the integration and utilisation of frameworks and models for investigating injury aetiology and mechanisms, and for developing athletic injury prevention strategies, is also presented.

Finally, it is important to acknowledge that ‘causality’ is a complicated metaphysical concept that remains the subject of many philosophical debates. Further, ‘causal inference’ is a complex and technical scientific and mathematical task that relies on triangulating evidence from multiple sources and on the application of a variety of methodological approaches [24–26]. While a deep dive into the philosophy and history of causality, and the vast array of methodological tools available for causal inference across the sciences, would certainly be of value, such a task requires a large body of work that is outside the scope of this article. Rather, this article simply highlights the necessity for causal knowledge within the context of athletic injury prevention, some of the major challenges facing the pursuit of causal knowledge in athletic injury research and prevention, and the value of graphical tools to the research process. In the current literature, various attempts to define causation have been presented [30], but a clear and agreed upon definition of causation continues to be elusive [25, 30], with some authors suggesting that the formation of such a definition may be too reductionist [25]. Accordingly, to avoid entering too deeply into the depths of the philosophy of causality, and for the operational purposes of this article, causality should tentatively be interpreted by its common understanding of cause and effect, i.e. the operation or relation of a cause and its effect. It should be noted that the terminology and language used in this article has been adopted from the (mutually compatible) causal inference [24–26] and sufficient component causal model [31–33] perspectives for understanding causality.

## The Four Step ‘Sequence of Prevention’ and ‘TRIPP’: A Brief Overview

**Fig. 1:**
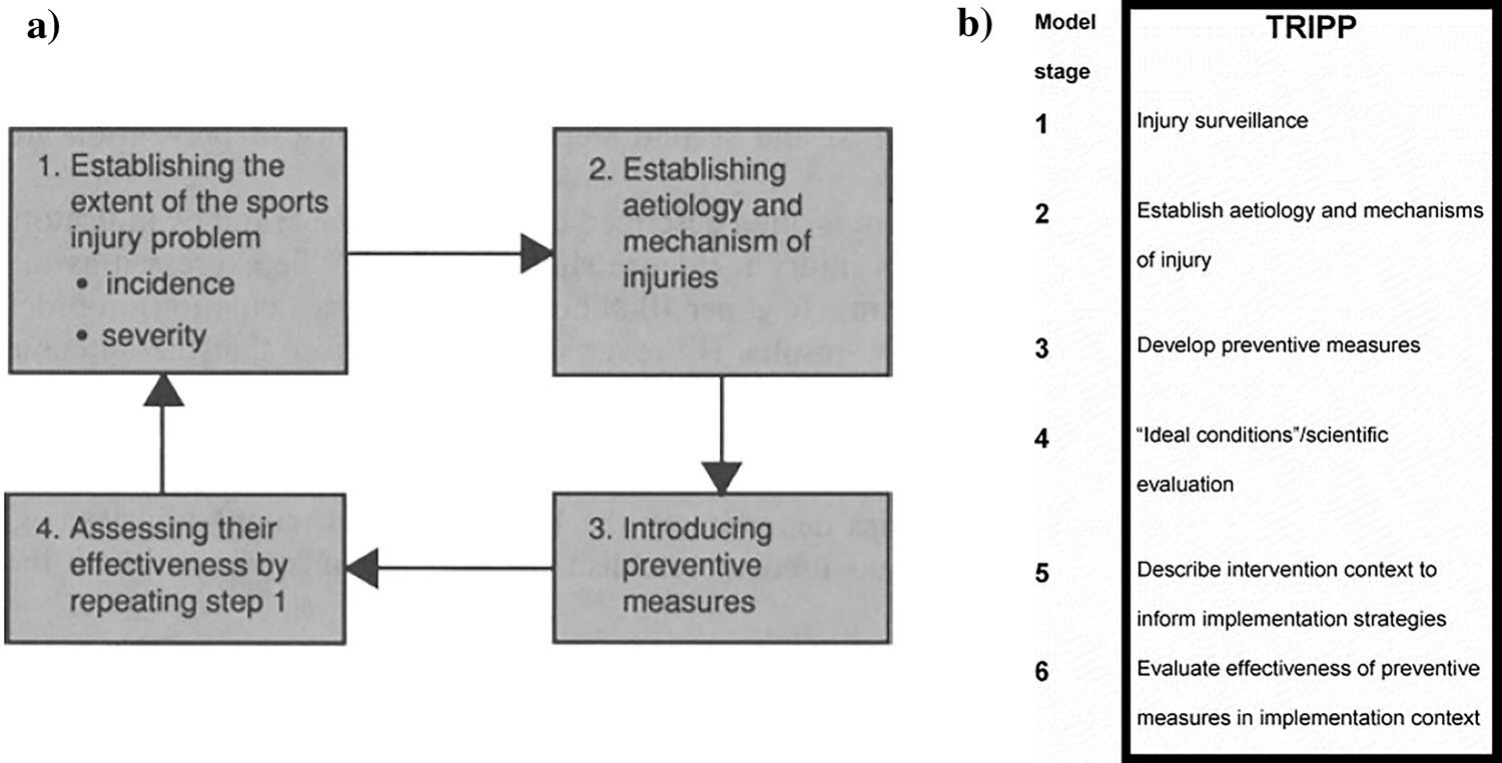
The ‘sequence of prevention’ (**a**) and the ‘Translating Research into Injury Prevention Practice (TRIPP)’ (**b**) models. Modified from van Mechelen et al. [12] and Finch [35] with permission

Injury prevention research has been described by van Mechelen et al. [12] as a four step sequence in the highly popularised ‘sequence of prevention’ (Fig. 1a). The ‘sequence of prevention’ is as follows. First, the magnitude of the problem should be identified and described in terms of the incidence and severity of sports injuries. Secondly, the risk factors and injury mechanisms that play a part in the occurrence of sports injuries should then be identified. The third step is to introduce measures that are likely to reduce the future risk and/or severity of sports injuries. Such measures should be founded on knowledge regarding the aetiological factors and injury mechanisms identified in the second step. Finally, the effect of the measures should be evaluated by repeating the first step, which can be achieved by time trend analysis of injury patterns or by means of a RCT [34]. Although various modifications and adaptations to this model have been proposed in literature, with arguably the most notable being the ‘Translating Research into Injury Prevention Practice framework’, or ‘TRIPP’ [35] (Fig. 1b), this article is primarily concerned with the establishment of injury aetiology and mechanisms and how to use this information to form and implement injury prevention strategies. Therefore, the current variations to this model and their added components will not be explored in detail here. However, their existence and contributions to the area are acknowledged. For further exploration on these models please see articles by van Mechelen et al. [12], Bahr and Krosshaug [36], and Finch [35].

## Establishing Injury Aetiology and Mechanisms: Current Challenges

### Variable Control and Randomised Controlled Trials in Athletic Injury Research

A key goal of science is to establish the causes of natural phenomena [13]. Causal knowledge provides the critical foundations on which informed and appropriate actions can take place to alter a given outcome, i.e. intervention. In the context of athletic injury, it is unsurprising then that a key feature of the van Mechelen ‘sequence of prevention’ [12] and Finch’s ‘TRIPP’ framework [35] is to introduce measures that are likely to reduce the future risk and/or severity of sports injuries on the basis of causal and mechanistic understandings. However, developing causal knowledge is a challenging endeavour. To develop causal knowledge, the controlled experiment is a mainstay of modern science, and for good reason. Although context dependent, it is generally maintained that, to estimate a causal effect, all major variables at baseline that can influence both the exposure and the outcome must be controlled for [24–26]. By holding constant all major variables influencing both the exposure and the outcome (with the exception of the independent variable of interest), one can determine if a particular variable is indeed responsible for a given outcome. However, achieving such conditions in real world settings can be extremely challenging. This is problematic. If a confounding (Table 1) variable remains uncontrolled for, this can lead to inaccurate conclusions about the causal relationship between variables (confounder bias). Concerns such as these gave rise to a particular type of controlled study, the RCT. In modern medicine, RCTs are commonly regarded as the ‘gold standard’ for assessing causality [37], as it is widely considered that the randomisation process applied to the samples within these types of studies serves to eliminate the effects of confounding variables [13, 37]. However, while RCTs certainly have high internal validity (Table 1) [38], the ‘gold standard’ label commonly attributed to this type of study remains controversial, as under many circumstances ethical or practical constraints oppose their implementation [25, 26, 38]. For example, in the absence of clinical equipoise (Table 1), it would be unethical to conduct an RCT that actively exposes one group of athletes to an intervention that is harmful (in relation to injury, performance or wellbeing). Notably, in some cases intervention may be physically impossible. For example, if we are interested in investigating the effect of either age or previous injury on injury risk, we cannot randomise individuals to different age groups or to having a previous injury. Thus, a RCT is not a feasible option for directly studying the causal effect of either of these variables on injury risk. It follows that, while RCTs certainly have high internal validity and should be conducted when feasible, the best method for understanding causality ultimately depends on what methods are actually available for answering a specific causal question [25, 26, 38].

Where RCTs are technically feasible in athletic injury research, there are also many practical challenges to overcome for the application of this particular type of study. At the forefront is obtaining a sufficient number of participants and injuries. This is crucial for achieving the necessary statistical power to ensure exchangeability (Table 1) between groups and to maintain the integrity of the RCT. However, this can be difficult, especially when a relatively small, potentially highly specialised population, such as elite athletes, is being investigated or when a specific type of injury is being studied. Indeed, conducting RCTs on elite sporting populations can be a particularly challenging endeavour, as not only are these populations limited in size, but owing to their professional nature, elite athletes and sports organisations may prove unwilling to cooperate and participate in studies that could potentially interfere with their training and preparation processes. As a result, RCTs are often conducted on alternative population groups, e.g. sub-elite athletes. Approaches such as these raise a number of concerns relating to the external validity and transportability (Table 1) of results when the findings from these studies are applied to different contexts, such as elite athletes [39]. While some of the practical considerations mentioned are not strictly insurmountable, these obstacles still pose significant challenges that researchers need to overcome to conduct this type of study successfully. Encouragingly, however, it is simply untrue that RCTs are the only way of discovering causality and developing causal knowledge [13, 25, 26]. Certainly, young children did not require a robust RCT to learn that putting their hand in the fire causes a painful burning sensation [13]. While RCTs are still widely considered to be the ‘gold standard’ for assessing causality [13] and should be done when feasible, exploration of alternative research approaches that may assist with the development of causal knowledge in relation to athletic injury appears necessary.

### Observational Research: Benefits and Pitfalls

Consistent struggles relating to ethical or practical constraints often restrict applied athletic injury research efforts and the appropriate application of RCTs. While RCTs are still widely considered to be the ‘gold standard’ for establishing causation, owing to their often-infeasible nature, especially at the elite sporting level, exploration of alternative methods and research practices that may assist with developing causal knowledge and guiding applied practice is warranted. In sports science, owing to their general simplicity and greater feasibility, descriptive research methodologies are frequently conducted to determine associations or correlations between variables, make predictions or describe injury risks and rates. These are useful studies that can provide the first exploratory step in the process of causality determination by identifying specific variables of interest. Across sports science, observational studies investigating the relationship between a selected variable of interest and athletic injury risk are particularly common. However, an important consideration when examining these types of studies and the potential relationships that may be present, is that association and correlation do not imply causation [13, 40]. This is a frequently reiterated mantra within the scientific community, and for good reason. Associations can present for a number of reasons other than causality, such as confounding [13, 24–26, 40].

**Fig. 2:**
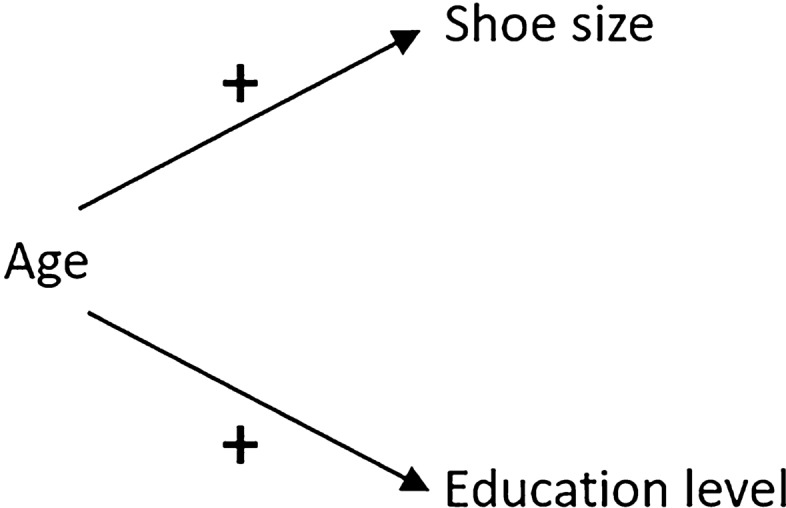
A signed causal DAG [62, 63] illustrating the basic structure of confounding: age is a common cause (confounder) of the relationship between shoe size and education level, producing a statistical relationship between shoe size and education level despite there being no causal relationship between these two variables. The (+) symbols represent a positive effect of age on shoe size and education level. *DAG* directed acyclic graph

To better highlight the issue of confounding, a simple non-sporting example of confounding is presented in Fig. 2. In this example, the causal relationships between three variables (age, shoe size and education level) in children are considered. Reasonably, it can be concluded that shoe size is not a cause of the level of education that a child receives, and the level of education a child receives is not a cause of a child’s shoe size, i.e. there is no causal link between these two variables. Despite this, shoe size and education level remain highly correlated in children. The reason for this correlation is that these two variables are statistically related by the effects of a third ‘confounding’ variable, age. In this example, age is considered to be a confounder as it is a common cause of both shoe size and education level (as children get older their feet grow and they go up school years). The consequence is that, owing to the effects of age on both shoe size and education level in children, shoe size and education level are statistically associated with one another despite there being no causal relation between them. Confounding is common in science, with observational studies being particularly susceptible to this form of bias. Accordingly, while observational studies may provide an estimate of the statistical association or correlation between variables, interpreting these relationships in a causal manner can be problematic and should only be undertaken after careful evaluation of the underlying assumptions.

### Confounding in Athletic Injury Research

#### Definitions of Athletic Injury

Confounding is a causal concept [25], and accordingly, prior to providing some examples of confounding in applied athletic injury research, it is worthwhile to explore some of the causal complexities and fundamental mechanisms that underpin athletic injury occurrence. It has previously been noted that injury occurs as a result of a transfer of kinetic energy to a tissue [36, 41, 42]. Athletic injury has also been described as occurring when the stresses and strains (Table 1) experienced by a tissue result in damage that is deemed severe enough to be considered an injury, i.e. tissue loading exceeds tissue strength [42, 43]. Note that these descriptions are, in fact, the same as one another, with the area under a stress–strain curve representing the energy absorbed during deformation, typically as a result of a transfer of kinetic energy. It follows that the International Olympic Committee (IOC) defines injury as “tissue damage or other derangement of normal physical function due to participation in sports, resulting from rapid or repetitive transfer of kinetic energy” [41]. While this is a useful definition, it is important to clarify some inaccuracies. Most notably, it is clear that damage or other derangement can exist without an injury necessarily being present. For example, muscle damage is common after sporting activity and may be an unavoidable part of the training process, while it can also be argued that fatigue constitutes a form of derangement of normal physical function. These should not be considered an injury. Rather, an injury more accurately occurs when the tissue damage experienced exceeds some critical damage threshold, whereby the damage sustained is not a normal part of the training process, but is rather chronically detrimental and severe enough to be considered an injury [43]. Future definitions of athletic injury should make such distinctions clearer.

#### The Ultimate Mechanism of Athletic Injury

**Fig. 3:**
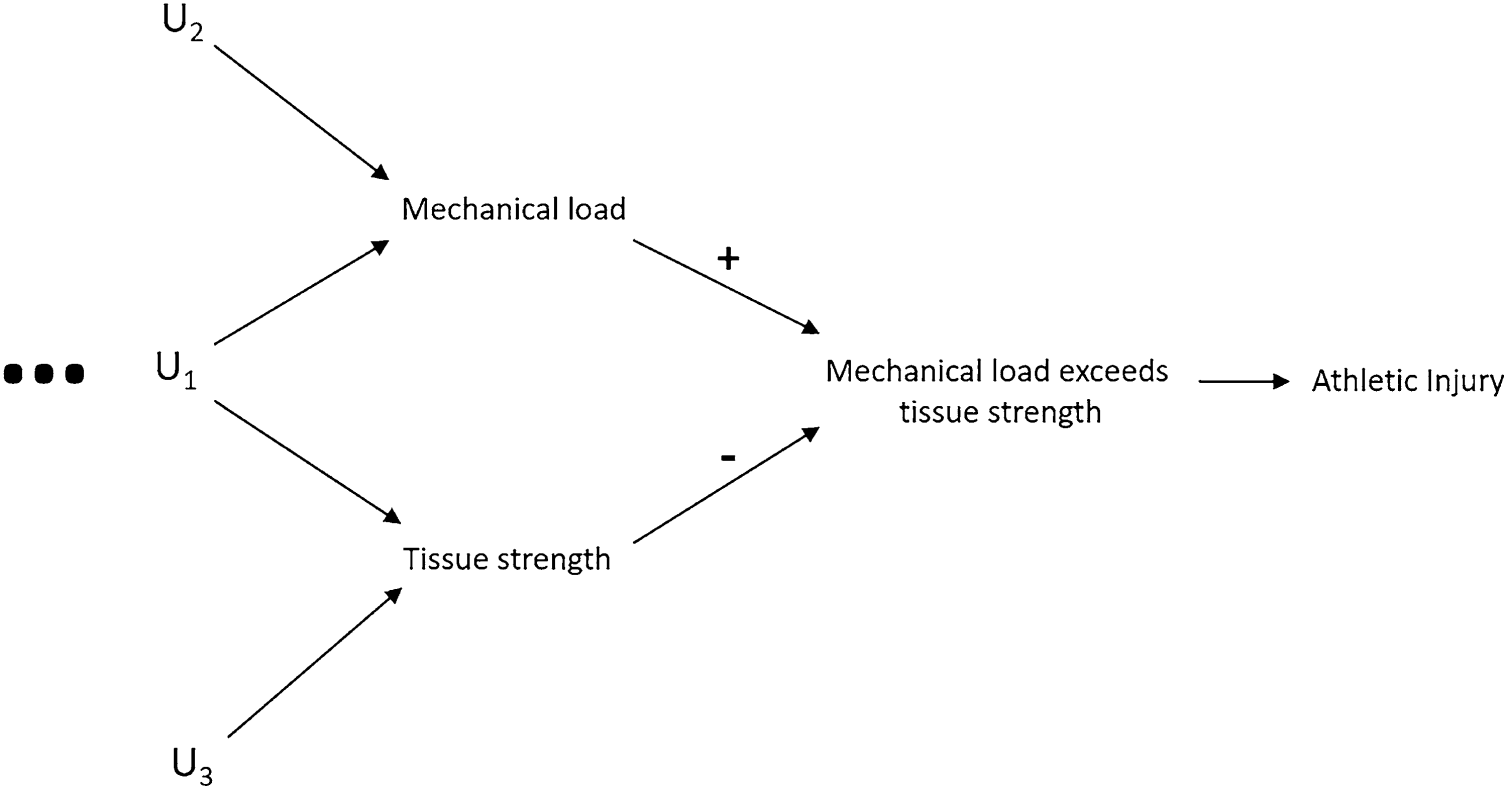
A signed causal DAG [62, 63] depicting mechanical loading exceeding tissue strength as the ultimate mechanism of acute athletic injury. A generic wider causal structure is included for illustrative purposes with the ‘U’ variables representing unspecified variables and the ellipses alluding to the existence of a wider causal network beyond that presented in the DAG. The (+) symbol reflects that as the mechanical load increases, the probability of the load exceeding the tissue strength and causing injury also increases. The (−) symbol reflects that higher tissue strength reduces the probability of the mechanical load exceeding this strength, decreasing the chance of injury. *DAG* directed acyclic graph

**Fig. 4:**
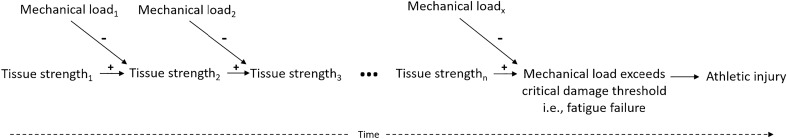
A signed causal DAG [62, 63] depicting repetitive mechanical loading as a cause of the deterioration of tissue strength (mechanical fatigue) and which eventually results in gradual onset injury occurrence. The subscript values represent the sequence of events, e.g. the tissue of interest has its original strength (tissue strength_1_) prior to a mechanical load (_1_) being applied. This causes a reduction in tissue strength (_2_) owing to mechanical fatigue. A second subsequent mechanical load (_2_) is applied, which causes a further reduction in tissue strength (_3_). This process continues until a critical damage threshold is exceeded and injury occurs. The (−) symbols represent a negative effect of mechanical loading on tissue strength (i.e. a reduction in tissue strength). The (+) symbols reflect that a higher initial tissue strength (e.g. tissue strength_1_) leads to a higher subsequent tissue strength (e.g. tissue strength_2_) despite repetitive mechanical loading reducing tissue strength over time. The ellipses in the figure represent a time jump to the point where injury occurs. Note: Physiological processes, i.e. remodelling and repair, have been intentionally omitted from this DAG for simplicity. *DAG* directed acyclic graph

Considering the definitions of athletic injury presented above, and as highlighted within a recently presented conceptual framework for athletic injury [42], the causes of an athletic injury can conceptually be separated into two fundamental components: the mechanical loading (force) experienced by a tissue and the mechanical strength (Table 1) of that tissue, one (or both) of which all causal variables must act through, i.e. these two components are reflective of the proximate (ultimate) mechanism of athletic injury. For illustrative purposes, two causal DAGs displaying this assumption for both acute and gradual onset injury in athletes are presented in Figs. 3 and 4.

In Fig. 3, a causal DAG is presented that is reflective of acute sudden onset athletic injury, whereby a single mechanical load exceeds the strength of a tissue. Some examples of this include anterior cruciate ligament rupture owing to sudden knee trauma, tibial break owing to a poorly timed tackle in soccer etc. In this DAG, the exceeding of tissue strength by a single mechanical load serves as the ultimate mechanism of injury. The ‘U’ variables presented in the diagram represent unspecified parent nodes (distal variables) of the ultimate mechanism and are included to represent a wider causal structure for athletic injury. This wider causal structure may be extensive and will vary between each individual injury type owing to unique, differing etiological pathways. In Fig. 4, a simple (wider variables excluded) causal DAG for gradual onset injury is presented, assuming no common causes of mechanical load, tissue strength and injury. Under this same assumption, an example of this type of injury may include a tibial stress fracture owing to repetitive loads from running. In this figure, repetitive mechanical loads fatigue a tissue until a critical damage threshold is exceeded and an injury occurs. In this type of injury physiological processes can also affect tissue strength, e.g. through remodelling and repair processes, but for simplicity’s sake this has intentionally been excluded from the DAG. In Fig. 4, the mechanical strength of the tissue is considered a time-varying variable as it deteriorates over time owing to cyclic loading. It is for this reason that tissue strength after each separate applied mechanical load is considered a different variable to tissue strength prior to that load; after each mechanical load is applied, tissue strength is represented by a new separate node.

#### Confounding in Athletic Injury Research

Within sports science and medicine research, many observational studies that assess relationships with and describe injury risks have been conducted across the sporting landscape, with a number of injury prevention recommendations and strategies commonly (but often erroneously) arising from such studies. As these studies are observational in nature, they are particularly susceptible to the previously highlighted issue of confounding. Accordingly, in the absence of appropriate supporting causal inference tools and structures, approaches such as these do not offer a reliable means for acquiring the necessary causal knowledge needed to intervene and alter injury outcomes.

**Fig. 5:**
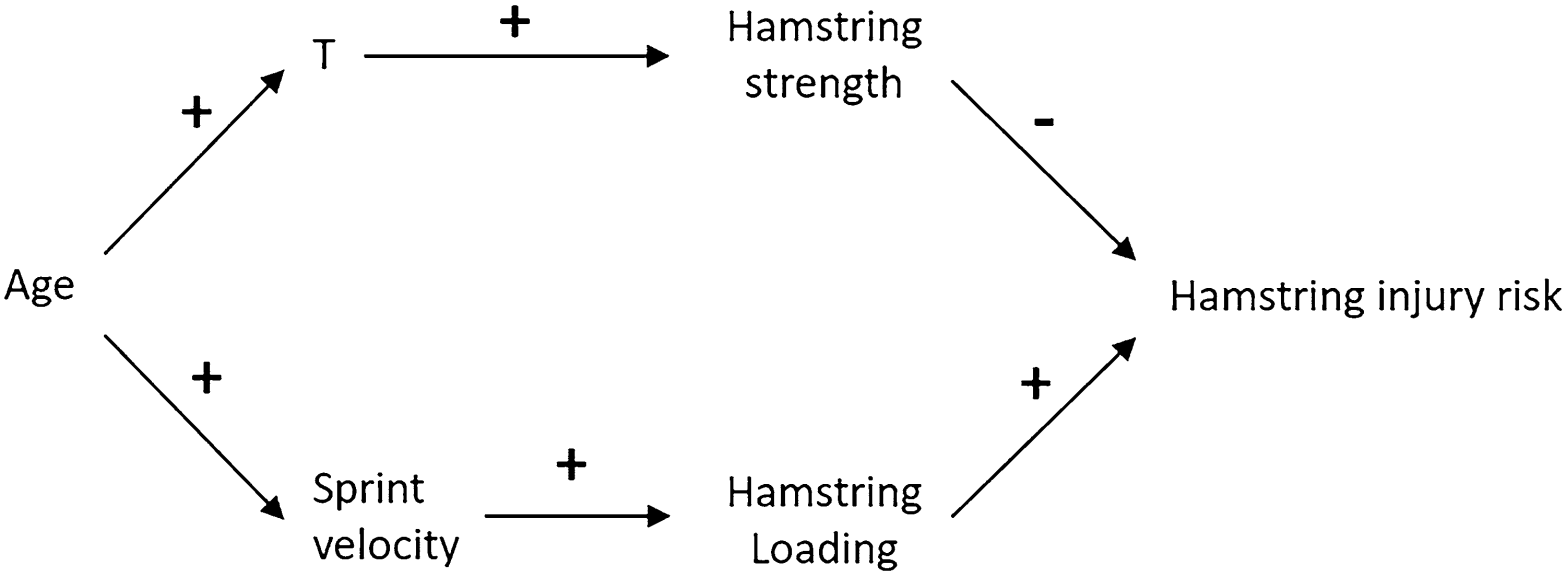
A causal DAG illustrating that age has a causal effect on hamstring injury through two identified causal pathways: (1) T, which has a protective effect on hamstring injury risk by increasing hamstring strength, and (2) increased hamstring stresses owing to faster sprint speeds (elite adult soccer players sprint faster than junior soccer players), which have a negative effect on hamstring injury risk. The (+/−) symbols represent a positive (increasing) and negative (decreasing) effect, e.g. the (−) symbol illustrates a reduction in hamstring injury risk owing to increased hamstring strength. Note: This is a simple DAG that is presented for illustrative purposes and should not interpreted as a complete example. *DAG* directed acyclic graph

To illustrate some of the problems that arise when using observational studies for estimating causal effects in relation to athletic injury, a contextually relevant example of confounding may prove useful. For instance, consider a theoretical scenario in which a researcher adopts an observational approach to investigate the relationship (association) between some hamstring tissue characteristic, labelled ‘T’, and the risk of hamstring injury in soccer players. For the purpose of this example, let us stipulate that (1) the data utilised to assess this relationship are derived from a large pool of soccer players of varying ages, including both elite and junior players; (2) T is some developmental characteristic, i.e. it increases with maturation into adulthood, and accordingly, elite level soccer players exhibit greater levels of T compared with junior level soccer players; and (3) greater levels of T also have a protective causal effect on hamstrings by increasing the mechanical strength of the hamstring muscles, but have no causal effect on athletic performance. Accordingly, in this scenario age has a beneficial causal effect on injury risk by increasing levels of T. However, age can also influence injury risk through other causal pathways. For example, elite adult soccer players sprint faster than junior soccer players, which subsequently increases the stresses placed on the hamstrings [44, 45]. Reasonably, this may increase hamstring injury risk. The nature of these proposed relationships is depicted in Fig. 5.

As shown in Fig. 5, age has an effect on hamstring injury through two causal pathways: (1) T, which has a protective (decreasing) effect on hamstring injury risk by increasing hamstring strength, and (2) increased hamstring stresses owing to faster sprint speeds (elite adult soccer players sprint faster than junior soccer players), which has an increasing (harmful) effect on hamstring injury risk. In this scenario, age is a common cause (confounder) of both T and the mechanical stresses placed on the hamstring. Therefore, the association that presents between T, our exposure of interest, and the risk of hamstring injury, our outcome of interest, will not be reflective of the causal relationship between these two variables, as this relationship will be impacted by confounding. This is important, since if higher levels of T are primarily associated with elite adult soccer players, and elite adult soccer players exhibit a heightened risk of hamstring injury (which indeed appears to be the case [46]), this may result in increased levels of T being statistically associated with an increased risk of hamstring injury despite actually having a causally protective effect. In scenarios such as this, causal interpretations of these associations may have dire consequences. Protective variables, such as increased levels of T, may be interpreted as being harmful to an athlete’s injury outcomes, while harmful variables, such as decreased levels of T, may be interpreted as being beneficial, i.e. causality is misinterpreted. Concerningly, in some contexts misinterpretations of causality will likely facilitate the implementation of inappropriate strategies that actively detrain athletes or damage athletic pursuits in an attempt to improve injury risk [9], and in some circumstances, may potentially even increase injury risk, e.g. implementing strategies that reduce levels of T as a result of T being associated with an increased injury risk when increased levels of T actually have a protective causal effect.

Alone, observational studies exploring relationships with injury risk cannot be expected to reliably bestow the necessary causal knowledge required to develop strategies to manipulate an athlete’s injury risk. Indeed, such is the potential impact of confounding on research outcomes, confounding can result in no association when there is a causal effect, an association when there is no causal effect and in some circumstances, associations that are in the opposite direction of the actual causal effect, such as in the example provided in Fig. 5. While the utilisation of observational studies for causal inferences is problematic owing to bias (‘association or correlation does not imply causation’), it is important to note that ‘some correlations do imply causation’, but appropriate causal inference tools and structures are needed [25]. Indeed, various tools exist that can assist with providing proposed causal structures, identifying and controlling for confounding and other forms of bias, as well as obtaining causal effects and knowledge from observational research, with this article being particularly concerned with introducing the reader to the importance of causal frameworks, models and DAGs in particular.

## Frameworks, Models and DAGs: Organising Ideas for Understanding Causation

Owing to the many challenges in athletic injury research and the potential pitfalls accompanying attempts to acquire causal knowledge from observational studies, there is a need to explore potential strategies that can assist with developing causal understandings of athletic injury. Notably, the formation and utilisation of frameworks, models and causal DAGs provides an approach that may alleviate some of these current obstacles and provide value for research pursuits.

### Frameworks and Models

In science, a framework serves as an intellectual structure (often graphically represented) that is used to make conceptual distinctions and organise ideas, deconstructing complex phenomena into relevant theories, assumptions, causal links and concepts of interest. In this respect, frameworks break down a system into relevant components, and align these components within a proposed theoretical, conceptual and/or causal architecture. Through this, frameworks help orientate research, with the adopted ideas and concepts assisting with the development and investigation of various research problems/questions, hypotheses, assumptions and causal links, while also serving as guides for collecting and analysing data and for interpretations of causality. Considering these benefits, it is unsurprising that some researchers have argued that, in the absence of a framework, research and scholarship are considerably ‘weakened’ [47, 48]. A variety of framework types are recognised across science, such as conceptual, theoretical and practical (Table 1), each distinguished by its unique functions and objectives [20].

While a scientific theory (Table 1) provides a substantiated explanation of a phenomenon in the natural world, and is used to make predictions that are testable by experiments or observations [47], a framework has the unique capacity to weave together multiple theories by providing a context within which the theories apply. Theories that might seem disparate at first can be interlinked within a framework that highlights their relevance to one another, and to a larger body of knowledge. Notably, when researchers contemplate how the various elements within a framework interrelate, they engage in theorising, i.e. form conjectures about the relationships between the concepts identified for a particular phenomenon. This process involves crafting hypotheses about the interplay among the concepts delineated for a given phenomenon, enhancing our understanding of the phenomenon and potentially leading to new scientific insights.

In contrast to theories and frameworks, models can be conceptualised as instantiations of theories or frameworks, and are therefore typically narrower in scope. Models may focus on a particular component of a given theory or framework, highlighting key elements, properties, and relationships within that component [14, 21]. Accordingly, models typically provide a more local description or understanding of a phenomenon, and commonly serve as intermediaries between theories and the real world [14]. However, owing to their more focused nature, models may also ignore a large part of reality. This is considered a feature [14, 21]. Much in the same way that a map of Rome is useful because it ignores much of reality to help us navigate Rome, a model may be useful because it ignores the rest of a particular theory or framework, instead focusing on specific components of interest [14, 21].

**Fig. 6:**
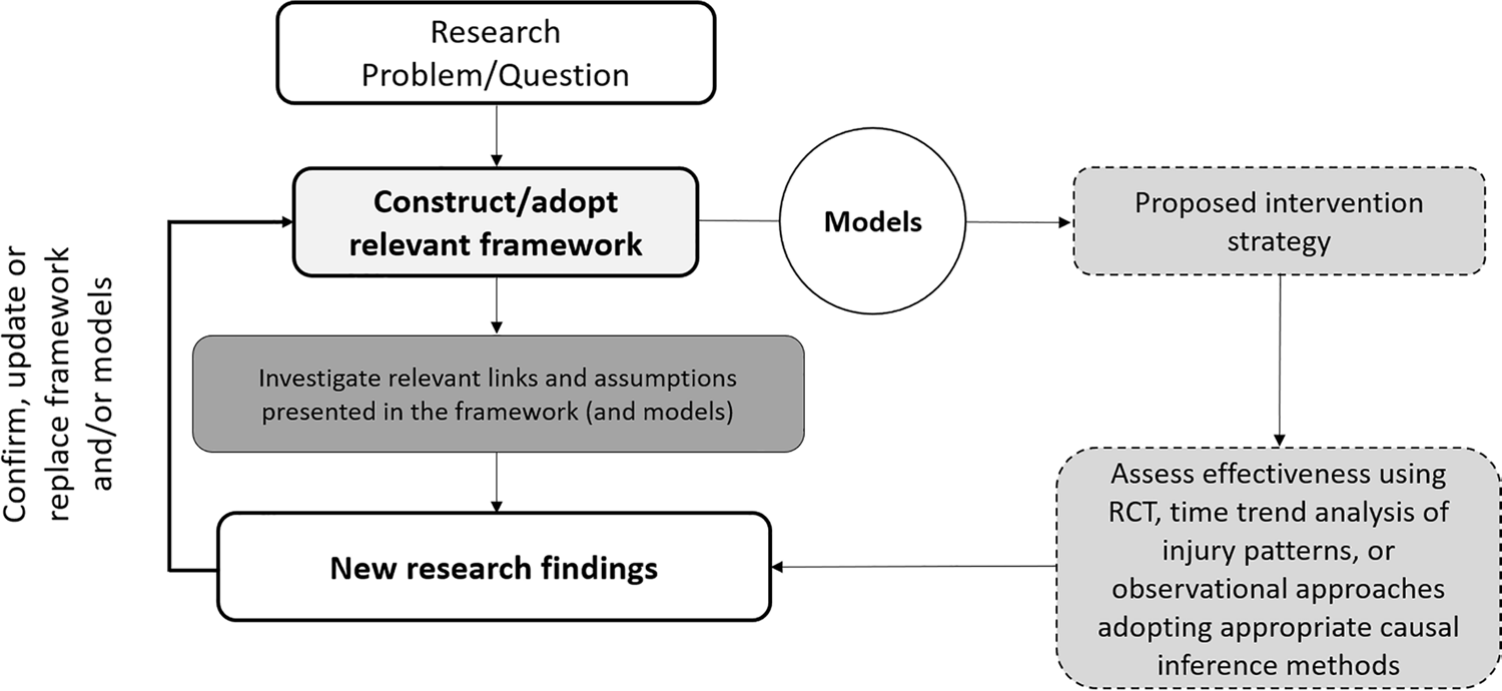
A proposed model for facilitating the integration of frameworks and models into the athletic injury research and prevention process

The utilisation and investigation of frameworks and models, and their proposed research links and assumptions, is akin to identifying, researching and confirming pieces of a puzzle with the intention of, one day, explaining the puzzle in its entirety. By deconstructing the problem into components, alternative scientific approaches and practices may be considered, and various elements and mechanisms may be targeted for investigation, potentially alleviating many of the challenges facing injury outcome-based research. Indeed, such an approach may be particularly useful for the translation of lab-based research into the applied sporting world, whereby the investigation of various causal links and assumptions within the controlled laboratory setting can better illuminate the specific roles of relevant variables and mechanisms within a given system. For example, as opposed to investigating the relationship between various tissue characteristics and athletic injury within the uncontrolled environment of the sporting arena, an alternative (and supplementary) approach that might offer important scientific insights could be to assess the relationship between these same tissue characteristics and tissue strength (under the assumption that tissue strength is a relevant mechanism through which various tissue characteristics can influence injury outcomes) within the controlled environment of the laboratory. Similarly, as opposed to researching the relationship between certain training load metrics of interest and athletic injury outcomes in applied sports settings, researchers can take an alternative approach. They could instead research how these training load metrics relate to tissue forces and cumulative tissue damage under the assumption that tissue forces and cumulative tissue damage are relevant mechanisms through which training load can cause certain types of athletic injury [9, 43, 49]. The knowledge gained from investigating specific research links in more controlled, and potentially more viable (depending on the context) environments can then inform the construction or revision of relevant frameworks. Researchers can then use the underlying theory and causal knowledge presented within these frameworks to develop useful multi-faceted models constructed with appropriate scientific reasoning for application in the applied sporting world, and to guide injury prevention strategies and decision-making. The effectiveness of these strategies can then be assessed by time trend analysis of injury patterns, observational studies using appropriate causal inference methodologies, or when possible, RCTs. Additionally, the comprehensive utilisation of frameworks and models can help guide the development of new, relevant technological advancements and metrics, while also ensuring that a coherent theoretical and conceptual underpinning is presented prior to the allocation of resources for resource intensive applied injury research studies (such as large-scale multicentre studies and RCTs). Accordingly, their adoption will assist with the development of appropriate conceptual foundations prior to the commencement of research studies, reducing resource wastage and minimising the risk of data fishing [16], p-hacking [16, 17] and HARK-ing [16, 18, 19]. Importantly, a framework can include a number of propositions, and accordingly, the various assumptions and links put forward within a framework should be challenged and either confirmed or dismissed through empirical evidence. Upon the emergence of data supporting new ideas and the subsequent dismissal of certain assumptions and links, a framework must be either revised or replaced, which is a normal step in the scientific process [20]. A proposal on how best to utilise frameworks and models for the investigation of injury causation and the development of injury intervention strategies is presented in Fig. 6.

### Causal Directed Acyclic Graphs (DAGs)

Causal DAGs, numerous examples of which have been presented throughout this article, are powerful tools in the science of causal inference. DAGs are a specific type of acyclic graph, i.e. there are no cycles, which provides a simple way of graphically representing, communicating and understanding key concepts of relevance regarding causality [23]. Specifically, DAGs provide a proposed causal model of reality, constructed by an investigator on the basis of their beliefs regarding the topology of the causal processes at work [25]. In this respect, DAGs require a subjective commitment by the investigator and can serve as a form of causal framework that explicitly lays out any underlying causal assumptions. This is an important feature that provides much needed transparency, as too often the causal assumptions of researchers are undeclared, and therefore unknown and unverifiable [26]. However, it is important to emphasise that DAGs are not valuable simply because they are explicit, nor are they solely conceptual in nature. DAGs are also complex mathematical tools, with each arrow between variables representing a quantifiable causal effect. It follows that, the development of DAGs has provided a critical step in the ‘mathematization’ of causal inference [25], and their value and importance in the science of causal inference should not be underestimated [24–26]. Indeed, these diagrams have opened up new avenues for understanding causality from observational research [24–26], as not only do DAGs provide a practical means of graphically representing specific causal pathways, mechanisms and assumptions, they also have important implications for guiding statistical analyses for the acquisition of causal effects from observational data. DAGs serve as potent instruments for addressing bias, explicitly showcasing critical confounders [and deconfounders (Table 1)], as well as highlighting when inappropriately adjusting for a variable will introduce new bias into the analysis, e.g. collider-stratification bias. As testament to their potency, a number of notable research successes can be attributed to DAGs, with these diagrams providing explanations for a series of apparent paradoxes, including the so-called Berkson’s [50], birth weight [51], obesity [52] and Simpson’s [53] paradoxes.

#### Causal Paths and Mediation (Mechanisms)

**Fig. 7:**
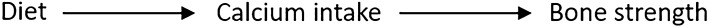
A causal DAG presenting the basic structure of mediation (Table 1). In this DAG, the effect of diet on bone strength is mediated by calcium intake, i.e. calcium intake is a mediator. *DAG* directed acyclic graph

A major benefit of causal DAGs is the explicit nature in which these diagrams outline specific causal pathways. In any complex system, numerous causal pathways may exist. In DAGs, a causal path is represented by a sequence of arrows (edges) that connect a set of variables (nodes), indicating the direction of causal influence from one variable to another. These paths represent hypotheses about how changes in one variable might propagate through a system to affect another variable. The identification of a causal path in a DAG can be quite straightforward. For example, in Fig. 5, it is quite clear that there are two hypothesised causal pathways through which age can impact hamstring injury risk. The identification of causal pathways is important for a number of reasons; it provides clarity in understanding direct and indirect effects (Table 1) within a causal relationship [a key feature of causal mediation analysis (Table 1)], enhances the accuracy of statistical models in predicting outcomes, and guides the development of more effective interventions and policies. Furthermore, it plays a critical role in identifying instances where an effect is mediated through another variable [a mediator (Table 1)]. In science, mediators are considered to be the mechanism that transmits the effect of one variable on another [25], and are visually depicted in a DAG as a variable that lies on the causal path between two variables. A simple example of this can be observed in Fig. 7, whereby calcium intake is an identified mechanism through which diet impacts bone strength.

While effective treatments have been produced in the absence of identified mechanisms, the identification of mechanisms is invaluable to science, as understanding mechanisms is critical for guiding interventions under changing conditions [25]. For example, in scenarios where bone strength is a concern and dietary consumption of calcium from food sources is lacking, understanding calcium intake as a key mechanism through which diet can impact bone strength can be vital for guiding the development and implementation of appropriate intervention strategies, such as additional calcium supplementation. Of course, many mechanisms can exist within a causal path. For example, in Fig. 7, one might identify calcium absorption as an important mediator of the effect of calcium intake on bone strength, and this could be added to the DAG. Nutrition is an area of science that offers many examples highlighting the importance of understanding mechanisms. In the late 1800s and early 1900s, had it been known that vitamin C was the mechanism through which citrus fruit worked to prevent scurvy, many sailors would not have lost their lives. Indeed, oranges and lemons would never have been replaced with cheaper alternatives (such as West Indian limes) that contained a fraction of the vitamin C [25]. Further, sailors would not have taken to boiling these fruits, degrading the vitamin C within them and disabling the mechanism through which fruits prevented scurvy. Subsequently, had it been known that vitamin C was the mechanism through which certain fruits prevented scurvy, this also would have prevented the role of citrus fruits in preventing scurvy being brought into disrepute [25].

#### Back-Door Criterion

**Fig. 8:**
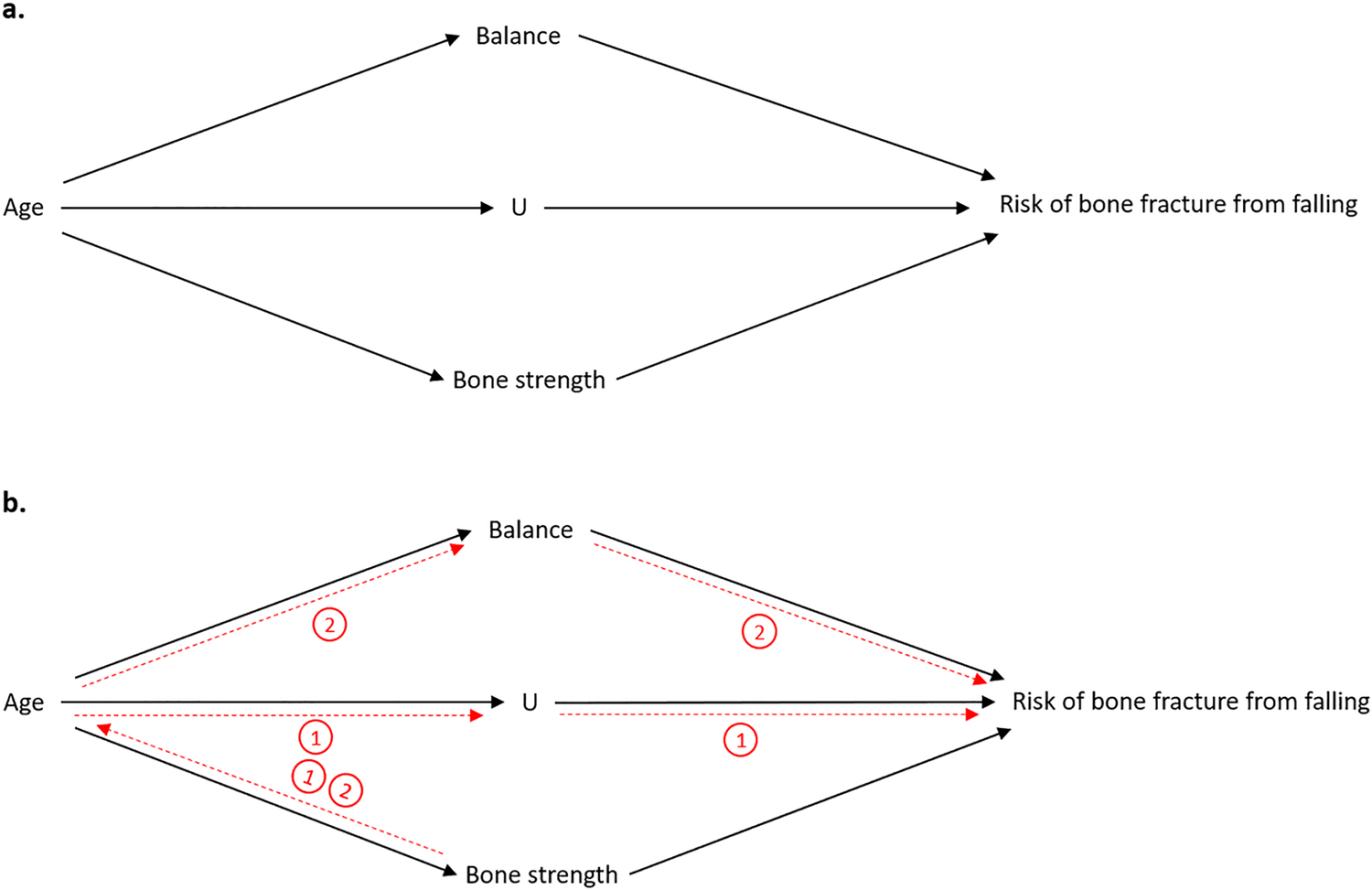
Back-door paths. Note: This is a simplistic DAG that is presented for illustrative purposes and should not be interpreted as a genuine proposal for risk of bone fracture from falling (RBFFF). In this scenario, bone strength is our exposure variable, and RBFFF is our outcome variable of interest. The red lines and numbered circles in **b** represent back-door paths (paths 1 and 2) between bone strength and RBFFF, which reveals confounding, i.e. which variables to control for in the statistical analysis to reveal the true causal effect of bone strength on RBFFF. Note that DAGs are typically presented as in **a**. It is abnormal to explicitly illustrate back-door paths in DAGs. The red lines are included in **b** for illustrative purposes. *DAG* directed acyclic graph

The back-door criterion is a fundamental concept in causal inference, providing a systematic method to identify and address confounding. This is achieved by identifying a set of variables that, when controlled for, block all ‘back-door paths’ (Table 1) from a treatment or exposure variable to the outcome variable of interest. A back-door path refers to any path from the treatment (or exposure) variable to the outcome variable that goes through a confounder. Accordingly, back-door paths are indicative of confounding and can create spurious (non-causal) associations between variables. The explicit nature of DAGs makes the identification of back-door paths, and by extent confounding, relatively straightforward. To exemplify this, a DAG displaying two back-door paths is presented in Fig. 8.

In the example presented in Fig. 8, bone strength is our exposure variable of interest, and risk of bone fracture from falling (RBFFF) is our outcome variable. In this example, we can see that there are two back-door paths (presented in red) between bone strength and RBFFF, both of which pass through age: path 1, which includes age and U (an unknown variable), and path 2, which includes age and balance. By identifying these paths, we now know which variables to control for in our analysis to reveal the true causal effect of bone strength on RBFFF (assuming that the DAG presented provides a valid representation of the world). As both back-door paths pass through age, the simplest solution to this problem would be to control for age, as doing so would close both back-door paths, i.e. by controlling for age we have controlled for all relevant bias in the DAG, and we can now determine the true causal effect of bone strength on RBFFF. However, let us assume that age is unavailable to us, and therefore cannot be controlled for. An alternative solution to this problem would be to control for the other variables along these back-door paths, as long as doing so does not introduce new bias into the analysis. By controlling for U, back-door path 1 is closed, but back-door path 2 remains open. Accordingly, to control for all of the bias, balance must also be controlled for to close back-door path 2. Once both back-door paths are closed, all of the bias is controlled for. Unfortunately, as U is an unknown variable in this example, we might not have a measure for it, which is a problem if we want to control for it. While there are certainly scenarios where controlling for all relevant confounding and bias using the back-door criterion is simply not possible, where the back-door adjustment fails, other methods not explored in this article are available and may offer alternative solutions, e.g. front-door adjustment, instrumental variables, Do-calculus, G-methods etc. [24–26].

#### Collider-Stratification Bias

**Fig. 9:**
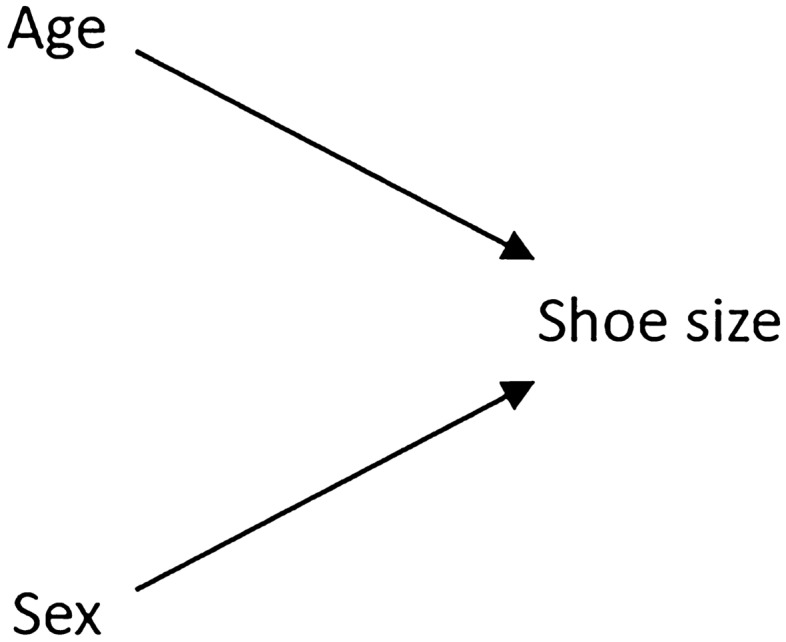
A causal DAG presenting the basic structure of a collider. In this DAG, age and biological sex are independent causes of a child’s shoe size, and shoe size is a collider. *DAG* directed acyclic graph

Confounding has featured quite prominently throughout this article, with its most basic structure being presented in Fig. 2. While confounding is a relatively well-known problem in research, a more commonly overlooked problem is that of ‘collider bias’, a form of overcontrol. Collider bias, in many ways, presents the opposite problem to confounding. In confounding, owing to the effects of a third confounding variable, two variables can start out statistically associated with one another despite actually being causally independent, i.e. there is a statistical relationship between two variables despite there being no causal relationship between these variables. In collider bias, two causally independent variables can start out statistically independent of one another, but by incorrectly controlling for a third (collider) variable, a spurious (non-causal) association is produced. To better illustrate this issue, a simple example of ‘collider bias’ is presented in Fig. 9. For this example, the shoe size and school children example presented in Fig. 2 is revisited, except in this case the causal relationships between age, shoe size and a new variable, biological sex, are considered. On the basis of our causal understandings of the relationships between these variables, it can reasonably be concluded that a child’s age is not a cause of a child’s biological sex, and biological sex is not a cause of a child’s age, i.e. there is no causal link between these two variables. However, both age and biological sex are causes of a child’s shoe size, i.e. as a child gets older their shoe size increases, and biological males tend to have larger feet than biological females as they mature [54]. This proposed causal structure is visually depicted in the DAG presented in Fig. 9.

In Fig. 9, it is clear that there is no causal relationship between age and biological sex, as indicated by the absence of an arrow between these variables. However, both age and biological sex have a causal effect on shoe size. In this example, shoe size is a collider (Table 1; illustrated by the two arrows ‘colliding’ at this variable). In causal inference, conditioning on a collider is disastrous if one’s aim is to find the causal effect between two variables, as doing so distorts the estimates of associations (collider bias). For example, if we were to control for shoe size (the collider) when analysing the relationship between age and biological sex, a new bias will be introduced into our analysis (collider bias), and age and biological sex will become statistically associated despite there being no causal relationship between these two variables. In certain scenarios, collider-related bias can become even more pronounced, such as when the analysis is stratified on the basis of different levels of a collider (collider-stratification bias). For example, if we were to stratify our analysis by different shoe size (the collider) categories when analysing the relationship between age and biological sex, we would introduce collider-stratification bias into our analysis. While it is unlikely that one would stratify their analysis by shoe size in a study investigating the relationship between age and biological sex, there are a number of well-known examples where collider-stratification bias has not been so obvious. Indeed, the identification of collider-stratification bias has led to important developments in improving understandings of various phenomena. For example, this form of bias largely explains the association reported between postmenopausal hormone treatment and coronary heart disease [55], the birth weight paradox [51] and the obesity paradox [56].

#### A Message of Caution for the Implementation of Causal DAGs

While it should be noted that some previous calls to include DAGs in sports science and medicine research have been presented in literature [57], these have been bereft of information and a more detailed outlining appeared necessary to foster greater engagement with these particularly powerful research tools. However, it is also important to emphasise that the information provided in this article should not be considered comprehensive or sufficient for the practical implementation of causal DAGs. Despite its apparent simplicity, the use of causal DAGs in research includes nuances, and a deeper understanding can help avoid some common pitfalls [58]. In addition, DAGs have many functions and purposes beyond those that have been explored in this article. The aim of presenting this information was primarily to act as an intermediary between the reader and causal DAGs to encourage further engagement with, and in-depth study of, these diagrams and the science of causal inference. As such, individuals interested in applying these concepts are strongly advised to seek additional resources, engage with more detailed academic literature, and, if possible, consult with experts in the field to ensure accurate and responsible application of these methods in their research endeavours. For readers seeking a more comprehensive understanding of the science of causal inference and the utilisation of DAGs for causal research, the following seminal texts on this topic are recommended [24–26].

## A Call for Injury-Specific Causal Diagrams (Where Necessary)

Considering the value of frameworks and models to the research process, it is unsurprising that a series of conceptual frameworks and models for athletic injury have been presented within the literature [36, 42, 43, 59–61], with some notable examples including the comprehensive model for injury causation presented by Bahr and Krosshaug [36], the Edwards framework for modelling overuse injury as a mechanical fatigue phenomenon [43], the Kalkhoven framework for stress-related, strain-related and overuse athletic injury [42] and the Bolling model for contextual factors [60]. While these models and frameworks are certainly useful for the organisation of ideas surrounding athletic injury, and for communicating key concepts of relevance regarding athletic injury causality, these frameworks and models are generic in nature, addressing all injuries within a singular framework or model. Accordingly, many of these diagrams do not address the unique mechanisms and circumstances that contribute to different types of injury. Given the multifaceted and intricate nature of specific injury types, a more tailored approach to athletic injury research and prevention appears increasingly necessary.

To address this gap, and where researchers consider it necessary, the development of causal frameworks and models, including causal DAGs, that are tailored to specific injuries and sporting contexts, is encouraged. This will facilitate a more nuanced analysis and understanding of the complex causal networks underpinning specific types of athletic injury, offering a foundation for the development of more targeted injury prevention strategies. Indeed, to reliably intervene across varying contexts, an understanding of the specific causal effects, and ideally the pathways and mechanisms, of relevant variables contributing to athletic injury occurrence is needed. This necessitates the development of a coherent causal model that explicitly outlines all causal assumptions, thereby allowing these assumptions to be subject to evaluation. The construction of such models may prove particularly valuable in the context of complex injury events, which may occur in many different ways across different sports, and potentially within the same sport, indicating multiple sufficient causal sets (Table 1) for a given injury type.

## Conclusions

Causal knowledge of athletic injury provides the critical foundations on which appropriate injury prevention strategies should be developed. However, acquiring causal knowledge is challenging. To assist, it is recommended that athletic injury research and prevention efforts should shift their attention towards the formation, utilisation, investigation and, where appropriate, revision or replacement of theoretically sound and evidence-informed causal diagrams, including frameworks, models and causal DAGs. The adoption of these tools will assist the research process by shifting athletic injury research away from a predominantly model-blind observational approach at high risk of bias, and towards a more sophisticated analysis of complex causal networks. Specifically, the adoption of causal diagrams will help organise key ideas and concepts surrounding athletic injury causation within a clear causal structure, opening up new avenues for the investigation of specific causal links and assumptions with appropriate scientific methods; leading to a more accurate and comprehensive understanding of the mechanisms underpinning sports injury occurrence. The adoption of causal DAGs in particular will provide much needed transparency regarding the causal assumptions of investigators (which are too often undisclosed and potentially even unknown by the investigators themselves) and will assist with the acquisition of causal effects from observational research. By enhancing understandings of injury causality, causal diagrams will also better facilitate the formation of appropriate athletic injury prevention strategies for utilisation in the applied sporting world. Such strategies should ideally be grounded in causal and mechanistic reasoning. Finally, to advance our field it is recommended that athletic injury researchers, and all of sports science and medicine, should engage more closely with the growing science of causal inference, for which seminal texts exist [24–26].
